# Development of acute Pseudomonas aeruginosa and Acinetobacter baumannii lung mono-challenge models in mice using oropharyngeal aspiration

**DOI:** 10.1099/acmi.0.000860.v3

**Published:** 2024-11-21

**Authors:** Irene Jurado-Martín, Chaoying Ma, Nouran Rezk, Maite Sainz-Mejías, Yueran Hou, John A. Baugh, Siobhán McClean

**Affiliations:** 1 School of Biomedical and Biomolecular Science, University College Dublin, Dublin, Ireland; 2 UCD Conway Institute of Biomolecular & Biomedical Research, University College Dublin, Dublin, Ireland; 3 UCD School of Medicine, University College Dublin, Belfield, Dublin, Ireland

**Keywords:** *Acinetobacter baumannii*, mouse lung challenge model, oropharyngeal aspiration, *Pseudomonas aeruginosa*

## Abstract

Antimicrobial-resistant pathogens such as *Pseudomonas aeruginosa* and *Acinetobacter baumannii* can cause potentially fatal infections in susceptible individuals, with respiratory tract infections among the most common clinical presentations. The development of novel treatments or prophylactic interventions to combat these infections is urgently needed and requires robust, reliable animal models for their preclinical evaluation. In particular, the bacterial burden needs to be accurately determined before and after administration of the potential therapy under evaluation to quantify the effectiveness of the treatment. We provide two reliable, non-invasive murine acute lung challenge models with either *P. aeruginosa* or *A. baumannii* using an oropharyngeal aspiration technique, which has been widely overlooked in studies testing vaccines or treatments for these pathogens. Here, we show that this non-surgical technique to deliver suspensions into mouse lungs does not significantly impact animal welfare (based on welfare monitoring and weight) and allows uniform bilateral distribution of the bacterial dose, resulting in even bioburden in both lungs. The optimal timepoint for humane killing and organ harvest was 24 h after challenge for both pathogens, and at least 4×10^6^ and 10^7^ c.f.u. per mouse were needed to obtain a reproducible *P. aeruginosa* or *A. baumannii* bioburden, respectively. These mouse challenge models offer a valuable tool to assess therapeutic interventions against *P. aeruginosa* or *A. baumannii* infections.

## Data Summary

All data associated with this work are reported within the article and supplementary materials.

## Introduction

*Pseudomonas aeruginosa* and *Acinetobacter baumannii* are two Gram-negative, non-spore-forming, ubiquitous bacteria belonging to the γ-proteobacteria class. *P. aeruginosa* is a rod-shaped bacillus, motile and facultative aerobe, while *A. baumannii* is a plum-shaped coccobacillus, non-flagellated and strictly aerobic [1,2]. Both species are major opportunistic pathogens causing a broad range of nosocomial infections, of which respiratory tract infections (RTIs) are particularly prevalent. Indeed, both *P. aeruginosa* and *A. baumannii* are frequently identified as primary aetiologic agents in RTIs [3,5], making them critical targets for clinical management and infection control efforts. Worryingly, more than 40 and 35% of *P. aeruginosa*- and *A. baumannii*-associated deaths, respectively, are due to infections in the lower respiratory tract [3]. Although the mortality rates for *P. aeruginosa* and *A. baumannii* RTIs vary between countries, mortalities of up to 60 and 84% have been reported, respectively [6,7]. Moreover, *P. aeruginosa* is a well-known cause of chronic lung infections in intubated patients and people with cystic fibrosis (CF), non-CF bronchiectasis or chronic obstructive pulmonary disease. These infections are associated with accelerated decline in pulmonary function, increased frequency of exacerbations and higher rates of mortality in these patients [8,12].

Both *P. aeruginosa* and *A. baumannii* are widely recognized as emerging threats to public health and consequently classified among the ESKAPE pathogens (*Enterococcus faecium*, *Staphylococcus aureus*, *Klebsiella pneumoniae*, *A. baumannii*, *P. aeruginosa* and *Enterobacter* spp.), which are grouped together because they are critical multidrug-resistant bacteria for which effective treatments are badly needed [13]. Moreover, the US Centers for Disease Control and the World Health Organization have classified both pathogens as urgent or serious threats and critical or high-priority pathogens, respectively [14,16]. The assessment of the efficacy and safety of any new therapies requires the use of reliable preclinical animal models for reproducible determination of the bacterial burden before and after treatment with the test agent. Given that *P. aeruginosa* and *A. baumannii* are prevalent respiratory pathogens [3,5], lung challenge murine models are important [17,18]. The inoculation method can affect the reproducibility of the model, and bacterial dose and time of infection require experimental evaluation [19]. The most widely used method is intratracheal administration via tracheotomy due to its accuracy and reproducibility, as bacteria are directly delivered into the lungs [19,20]. However, the surgery is complicated and invasive, staggering the start times of the experiment and risking animal welfare due to the procedure. While environmental exposure and intranasal delivery are easy-to-perform, non-invasive methods that mimic the natural acquisition of bacteria [21,22], they require a higher number of animals due to their low reproducibility, as the actual number of bacteria that reach the lungs is ill-defined and may not colonize both lungs evenly [19,20]. Delivery via peroral cannulation is less invasive than tracheotomy and allows for precise bacterial dosing, but the procedure still carries the risk of airway trauma and requires a high skill level [19,20, 23]. Therefore, we aimed to adapt a method that was reproducible, with low physical and physiological impacts on the animal and low contamination of non-target tissue. A non-invasive, technically easy and rapid-performing oropharyngeal aspiration technique was reported by Lakatos *et al.,* which described the uniform delivery of silica within the lung [24]. A variation in this procedure was subsequently described by Madenspacher and Fessler to develop an acute *K. pneumoniae* pneumonia [23]. However, despite its benefits, this method has been infrequently used in the context of *P. aeruginosa* and *A. baumannii* challenge models and the evaluation of novel interventions. Our objective was to develop a reliable mouse challenge model that results in reproducible bioburden in the lungs without impacting animal welfare and thus extend the use of this approach. We established an acute lung mono-challenge model for either *P. aeruginosa* or *A. baumannii*, which was of mild severity and refined with minimal low impact on animal welfare and consequently in line with the 3Rs principle (reduce, refine and replace) [25]. Our findings offer guidance on how this model can be effectively employed, enabling other researchers to adapt and utilize it according to their specific experimental needs. This model has the potential to be a versatile tool in the study of these pathogens, facilitating the exploration of infection mechanisms and the evaluation of novel prophylactic/therapeutic strategies.

## Methods

### Bacterial strains

For the *P. aeruginosa* model, the clinical isolate KK1 from the international *P. aeruginosa* reference panel [26] was used. It was originally obtained from the sputum of a 16-year-old CF patient in Hannover (Germany), and it is the early strain from the KK series of sequential isolates (KK1, KK14 and KK72) [27]. It is a well-characterized strain with virulence traits that allow the colonization and infection of mouse lungs [28]. For the *A. baumannii* model, we utilized a well-characterized laboratory strain ATCC 19606 (LMG 1041), which was first isolated in the US from a urinary tract infection case in 1948 [29]. This strain was selected as it is one of the most extensively characterized strains and is frequently employed in studies focusing on virulence and vaccine development.

### Growth curve determination

Bacterial cultures were grown overnight in Luria-Bertani (LB) broth at 37 °C and 200 r.p.m. and re-inoculated into fresh LB broth (1 : 5 dilution for *P. aeruginosa* and 1 : 10 for *A. baumannii*). Optical density at 600 nm (OD_600_) measurements and samples were taken every 15–30 min until the stationary phase was reached. Samples were ten-fold serially diluted in PBS, plated onto LB agar in duplicate and incubated at 37 °C for 24 h, and the resulting colonies were counted to determine c.f.u. ml^−1^. Two to three independent experiments were performed. OD_600_ measurements were correlated with c.f.u. ml^−1^ to allow a strain-specific growth equation.

### Bacterial challenge

We employed C57BL/6 mice for the *P. aeruginosa* model, as these are widely used for *P. aeruginosa* challenge studies. For *A. baumannii*, we based our approach on a recent study that developed a sub-lethal pneumonia model in C3H/HeN mice [30]. In that study, the mice were intratracheally inoculated (via tracheotomy) with 1×10⁷ c.f.u. of *A. baumannii* ATCC 19606, resulting in 100% survival up to 14 days post-infection, with a sustained bacterial burden of 3-log_10_ c.f.u. per organ in their lungs. To further refine this model and incorporate the oropharyngeal aspiration technique, we continued to use C3H/HeN mice for *A. baumannii*. Female C57BL/6J and C3H/HeN mice (6–8 weeks old) were used to establish the *P. aeruginosa* and the *A. baumannii* models, respectively. Both mouse strains were purchased from Charles River (UK) and randomly grouped in three to four mice per cage. Food and water were available *ad libitum*. Fresh cultures of either strain were grown to OD_600_=0.6 as before, and aliquots were pelleted and resuspended in an equal amount of sterile PBS. The strain-specific growth equation was used to determine the appropriate amount of culture for the required bacterial dose (Table 1), and the inocula were diluted in PBS and plated onto LB agar in duplicate to confirm the c.f.u. administered.

**Table 1.** Bacterial doses and corresponding c.f.u. ranges used to optimize an acute lung infection model of *P. aeruginosa* or *A. baumannii* in mice

Dose range	c.f.u. 50 µl^−1^	
	*P. aeruginosa*	*A. baumannii*
Low	<5×10^5^	<1×10^6^
Medium	6×10^5^–5×10^6^	1x10^6^–9×10^6^
High	>6×10^6^	1×10^7^–9×10^7^

**Fig. 1.**
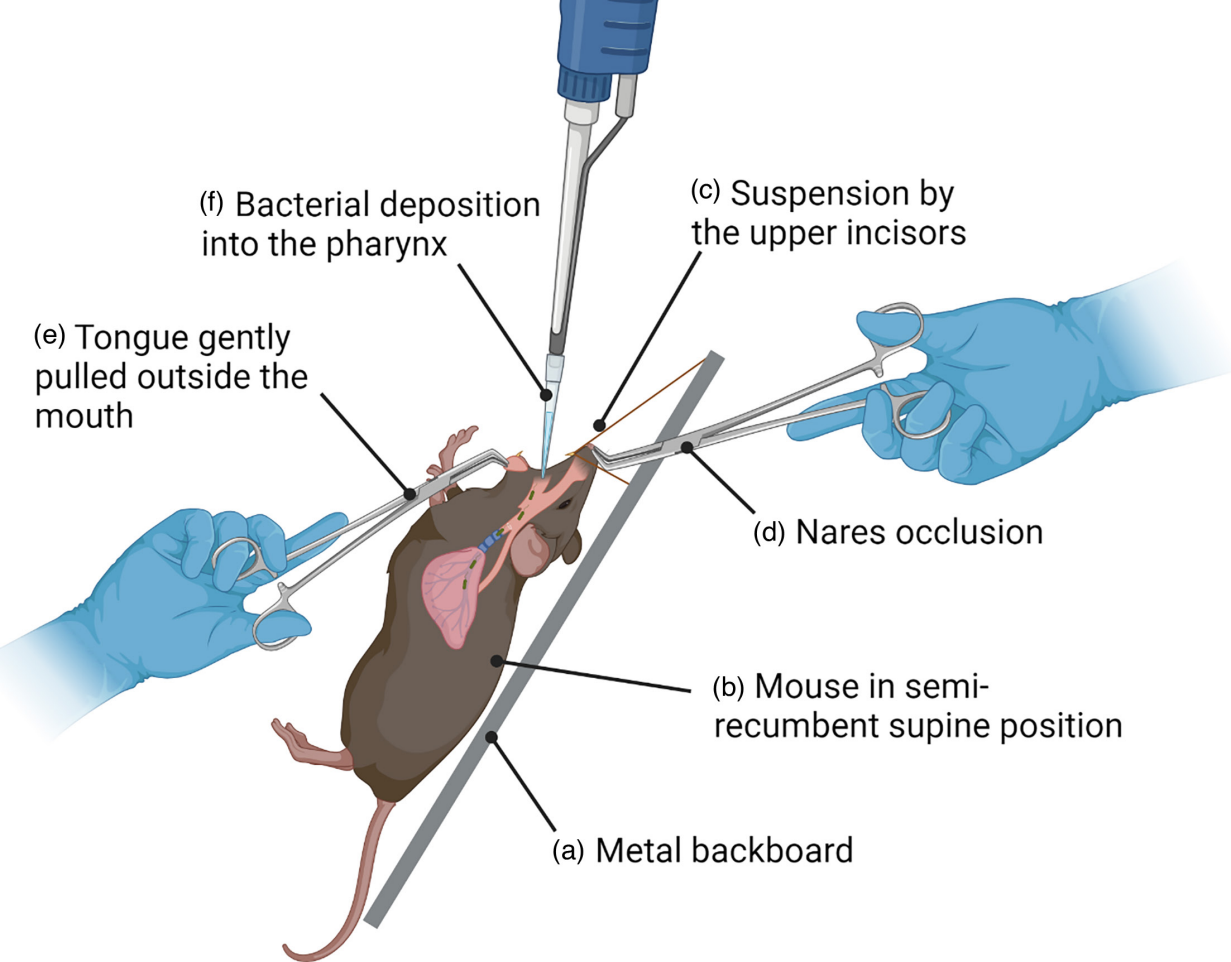
Diagram representing the oropharyngeal aspiration technique for the development of a lung challenge model in mice. Once anaesthetized, the mouse is placed on a metal backboard (**a**) in a semi-recumbent supine position (**b**) and suspended by the upper incisors from a thread (**c**). The nares are then occluded (**d**), and the tongue is gently pulled outside the mouth using blunt tweezers (**e**) by one person. An aliquot of 50 µl of either a bacterial suspension or PBS is deposited by the second person into the oral cavity (**f**) as close as possible to the entrance of the trachea with a P200 pipette and filter tips. The mouse is allowed to aspirate the inoculum for at least ten breaths, while respiration is monitored both visually and aurally, after which the tongue and the nares are released, and the mouse is allowed to recover in the cage’s bedding.

On day 0, mice were weighed and challenged with freshly prepared bacterial inocula using the oropharyngeal aspiration technique (Fig. 1) [23]. Mice were individually anaesthetized in a chamber with 5% isoflurane gas and 2 l min^−1^ oxygen for 1 min before the procedure. Once anaesthetized, mice were placed on a metal backboard in a semi-recumbent supine position and suspended by the upper incisors from a thread (Fig. 1). The nares were then occluded, and the tongue was gently pulled outside the mouth using blunt tweezers (Fig. 1). An aliquot of 50 µl of either a bacterial suspension or PBS was deposited into the oral cavity as close as possible to the entrance of the trachea using a micropipette and filter tips. Mice were allowed to aspirate the inoculum for at least ten breaths, while respiration was monitored both visually and aurally, after which the tongue and the nares were released, and mice were returned to their cage to recover. Mice were weighed and monitored daily after the challenge, using an experiment-specifically designed welfare scoresheet (Table S1, available in the online version of this article) that evaluates changes in respiration/position, behaviour, weight loss and hydration, combined with the mouse grimace scale (MGS). Any mice that reached a humane endpoint as defined in the welfare score sheet (Table S1) were euthanized.

### Bacterial burden determination

At the required timepoints, the mice were euthanized by CO_2_ exposure (2 l min^−1^) for 9 min plus a 5 min delay, confirming the death by sensing the absence of heartbeat, and then dissected in a biosafety cabinet as follows. Mice were fixed on a ventral position and disinfected with 70% industrial methylated spirit. All instruments were cleaned likewise between mice and organs. Both the dermis and epidermis were cut from below, up to the jaw; the thorax was opened by cutting the sternum, and the right and left lungs were removed. Spleens were collected to assess bacterial dissemination and invasiveness. Stomachs were also collected to evaluate bacteria being swallowed rather than inhaled [23]. Organs were individually placed onto 1 ml of sterile PBS and weighed before homogenization. Organs from mice were homogenized in the TissueLyser II (Qiagen) for 15 min at the maximum frequency (30 Hz s^−1^) using stainless-steel beads (3.2 mm, one per tube). Alternatively, organs were homogenized in a FastPrep-24™ (MP) homogenizer at 5 m s^−1^ for 60 s for spleens, 2 min for lungs and 5 min for stomachs. Homogenates were ten-fold serially diluted in PBS, plated onto LB agar in duplicate and incubated at 37 °C for 24 h, after the c.f.u. was counted and normalized to organ weight (c.f.u. g^−1^).

### Statistical analysis

Statistically significant differences (*P*<0.05) in the bacterial burden in the organs, severity scores or weight loss were analysed by one-way ANOVA using GraphPad Prism v 9.4.1.

## Results

### Development of an acute *P. aeruginosa* lung challenge model in mice

**Fig. 2.**
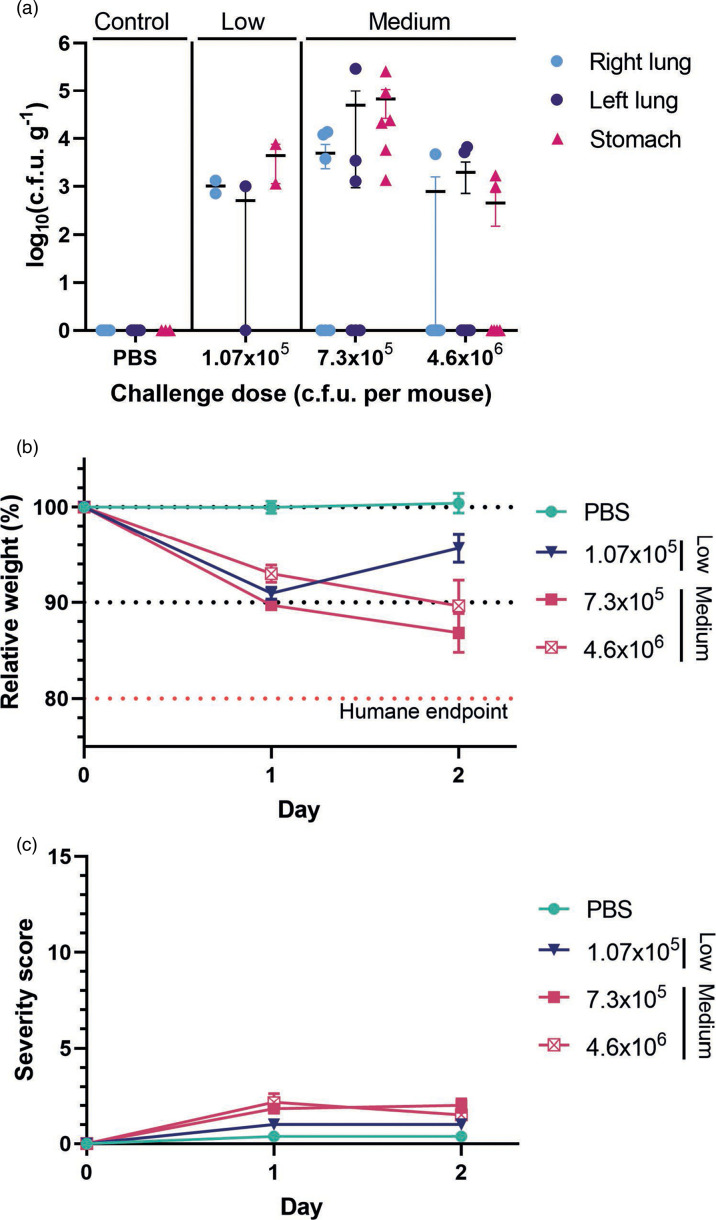
Optimization of *P. aeruginosa* dose and time in the development of the acute pneumonia model 48 h after challenge. (**a**) c.f.u. recovery showing the bioburden in the lungs and stomachs of mice challenged with PBS, low and medium bacterial doses. Bars represent the mean±sem, and each dot represents one mouse. (**b**) Changes in mice weight relative to that before the procedure. (**c**) Severity score reached after challenge. Red dashed line indicates the humane end point. (b, c) Each point represents the mean±sem of the mice in the group [PBS; low dose *n*=2 (dose-finding pilot study); medium doses *n*=6].

Non-lethal animal models to assess vaccines and antimicrobial treatments rely on accurate and reproducible evaluation of bacterial c.f.u. The first stage of development of the challenge model was determining the c.f.u. recovered from the right and left lungs and stomachs 48 h post-challenge with three bacterial dose ranges that are referred to as low-, medium- and high-dose ranges (Table 1). The medium-dose range resulted in bacterial burdens in the log 3 to log 5 c.f.u. g^−1^ range at 48 h post-challenge (Fig. 2a), while mice in the low *P. aeruginosa* dose (<5×10^5^ c.f.u. per mouse, a pilot dose-finding group) almost completely cleared the bacteria within 48 h of challenge (Fig. 2a), correlating with the weight recovery observed between days 1 and 2 (90.96 and 95.69 %, respectively) (Fig. 2b).

The severity of the procedure was measured on a scale of 0–15 based on behaviour, hydration, grimace scale (all 0–3) and binary observations (pain, wound and skin lesions) (Table S1). A key criterion in the development of this model was to identify an optimal and reproducible bacterial dose that did not exceed mild severity scores (a score of 7 or greater, Table S1). Mice receiving high bacterial doses (>6×10^6^ c.f.u. per mouse) reached humane end points and had to be humanely killed at 24 h. The mice in the low- and medium-dose groups showed low severity scores (Fig. 2c). Mice in both groups that received bacterial doses within the medium-dose range (6×10^5^ – 5×10^6^ c.f.u. per mouse) experienced weight loss over the 2 days post-challenge (the first group 89.71 and 86.85 %, and the second group 93.03 and 89.65 %) (Fig. 2b), with only a slight increase in severity scores observed (Fig. 2c). Despite this, the bioburden in the lung was low in both groups (log_10_ <1, and log_10_ <2, respectively) at 48 h, suggesting the clearance of the bacteria over this time. As expected, no bacteria were recovered from the organs of mice in the control group (Fig. 2a), and these mice did not experience weight loss nor showed signs of sickness (Fig. 2b, c).

**Fig. 3.**
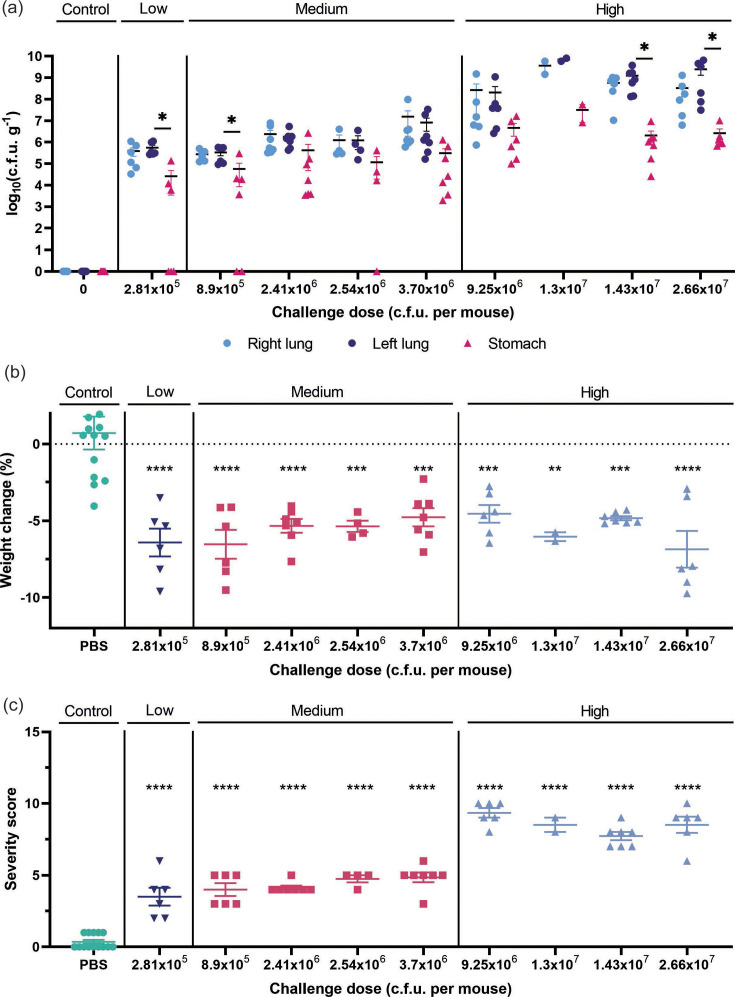
*P. aeruginosa* bioburden in the acute pneumonia model 24 h after challenge. (**a**) Bioburden in the lungs and stomach of mice challenged with PBS, low, medium or high bacterial doses. (**b**) Relative weight lost or gained by mice relative to that before the procedure. (**c**) Severity score reached after the challenge. In all graphs, results are represented with the mean±sem (*n*=6–7, except for two groups which were *n*=2 and *n*=4, as part of the initial dose-finding pilot study), with each point representing one mouse. Statistical differences between organs (**a**) or with the control group (**b, c**) were determined using one-way ANOVA (*P* value < 0.05) (*P ≤ 0.05; **P ≤ 0.01; ***P ≤ 0.001; ****P ≤ 0.0001).

**Fig. 4.**
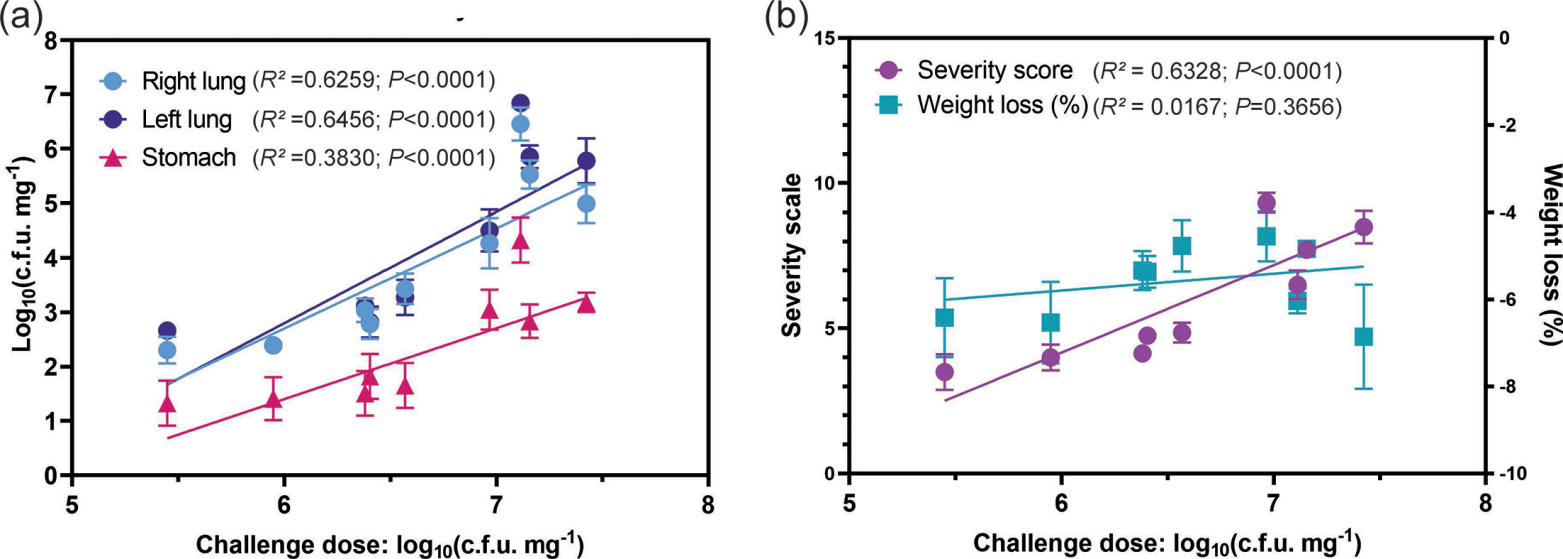
*P*. *aeruginosa* challenge dose correlates with c.f.u. recovered in the lungs and stomach (a) and the severity scores and weight loss, non-linear sigmoidal regressions (b) 24 h post-challenge, linear regression. Each point represents the mean±sem of the mice in the group. The corresponding *R*^2^ and *P* values of the correlations are displayed. These data represent three independent experiments.

Mice inoculated with low or medium bacterial doses showed higher bioburden in the lungs after 24 h than after 48 h (~2 log_10_ more). The c.f.u. recovered 24 h post-challenge from the lungs increased with the challenge dose, and both right and left lungs showed equivalent c.f.u., regardless of the bacterial inoculum administered (Fig. 3a). Linear correlation analysis showed a statistically significant positive correlation between the challenge dose and c.f.u. recovery 24 h after challenge, and these correlations are comparable for right and left lungs (Fig. 3b). As predicted, due to the nature of the inoculation technique, some c.f.u. recovery was also observed in the stomachs. In early experiments, this was always at least one log c.f.u. lower than that of the lungs (Fig. 3a); however, with proficiency, this reduced to less than 2% (Fig. S1). Linear correlations also confirmed that the bioburden in the stomach remained lower than that of the lungs (Fig. 4a). As expected, no bacteria were recovered in organs from mice in the control group (Fig. 3a). Importantly, the model was highly reproducible as administration of two challenge doses (2.41×10^6^ and 2.54×10^6^) in separate independent experiments resulted in a comparable bacterial bioburden. Therefore, our results show that the peak of bioburden was reached at a 24 h timepoint.

All the mice that were inoculated with bacteria, regardless of dose, lost weight by 24 h post-challenge, although weight change did not correlate with the challenge doses (Figs3b  4b). In contrast, the severity scores reached in mice challenged with high doses (8.51±0.66) were double than those challenged with medium or low doses (4.25±0.56) (Fig. 3). Moreover, there was a statistically significant positive correlation between the challenge dose and the severity score reached by the mice (Fig. 4b, *r*=0.6328). This suggests that the signs of sickness in mice due to *P. aeruginosa* infection may be better measured using parameters such as appearance, behaviour or the MGS, rather than weight loss. Overall, these studies indicate that organ collection at 24 h post-bacterial instillation and a challenge dose of at least 4×10^6^ c.f.u. per mouse of the *P. aeruginosa* KK1 strain were required to ensure enough bacterial burden in the organ without reaching a humane end point.

### Development of an acute *A. baumannii* lung challenge model in mice

**Fig. 5.**
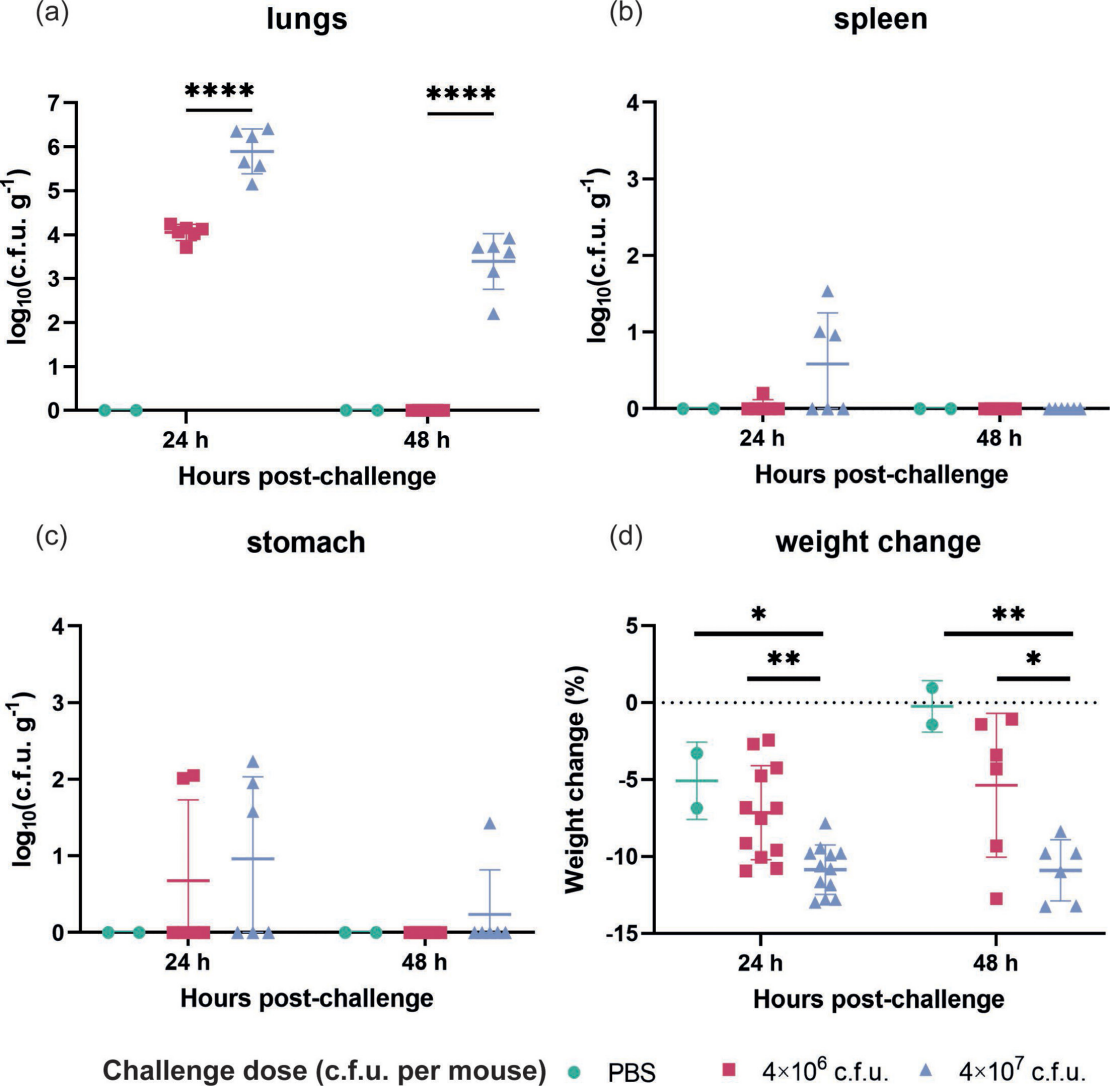
Optimization of the *A. baumannii* dose in the development of the acute pneumonia model at 24 and 48 h after challenge. Bacterial burdens in lungs (a), spleens (b) and stomachs (c) were presented as mean±sd. The limit of detection is 1 c.f.u. g−1, i.e. log (c.f.u. g−1) = 0. Body weight change at 24 and 48 h post-challenge (d) was presented as mean±sd. *n*=7 for high- and medium-dose groups; *n*=2 for PBS control. These data represent three independent experiments. Statistical differences were determined using one-way ANOVA (*P* value < 0.05) (*P ≤ 0.05; **P ≤ 0.01; ***P ≤ 0.001; ****P ≤ 0.0001).

The bacterial burdens in the lungs, spleens and stomachs of mice challenged with medium (4×10^6^ c.f.u.) and high (4×10^7^ c.f.u.) doses of *A. baumannii* ATCC 19606 were assessed at 24 and 48 h post-challenge. At 24 h, mice inoculated with the medium dose exhibited bacterial burdens in the lungs ranging between 3- and 5-log_10_ c.f.u. g^−1^. In contrast, the high-dose group demonstrated significantly higher burdens, ranging between 5- and 7-log_10_ c.f.u. g^−1^ (Fig. 5a). Statistically significant differences in bacterial burden were observed between the medium- and high-dose groups at both 24 and 48 h post-challenge (*P*<0.0001), underscoring the dose-dependent nature of the infection (Fig. 5a). Notably, by 48 h post-challenge, mice in the medium-dose group showed complete clearance of bacteria from their lungs, indicating that a medium-dose challenge is less robust. Conversely, the high-dose group retained a measurable bacterial burden at 48 h, although this had decreased relative to the 24 h timepoint, with values between 2- and 4-log_10_ c.f.u. g^−1^.

The bacterial dissemination to the spleen was minimal, as was its appearance in the stomach. At both timepoints, bacteria were largely confined to the lungs, with only low levels detected in the spleens and almost no c.f.u. present in the stomachs (Fig. 5b, c). The bacterial burden in these organs was consistently <1 log_10_ c.f.u. g^−1^, indicating the limited systemic spread of *A. baumannii* ATCC 19606.

In terms of host morbidity, all mice subjected to bacterial challenge experienced weight loss, although the magnitude of loss was moderate. The body weight of mice in the high-dose group dropped to 89.14% at 24 h post-challenge (*P*=0.0135, compared to PBS control). Interestingly, this level of weight loss (89.1%, *P*=0.0079 compared to PBS control) persisted at 48 h with no further significant decline, indicating that while the bacterial challenge induced some physiological stress, it did not result in severe systemic deterioration. The relatively mild weight loss, coupled with the bacterial clearance observed in the medium-dose group, suggests that the mice were undergoing a manageable level of suffering, with the bacterial burden being largely restricted to the lungs and not causing overwhelming systemic effects. Overall, studies indicated that organ collection at 24 h post-challenge was optimal and that at least 4×10^7^ c.f.u. per mouse of the *A. baumannii* ATCC 19606 strain is required to ensure sufficient and reliable bacterial burden in the organs without reaching a humane endpoint.

## Discussion

To test the efficacy of novel drugs or vaccines, it is necessary to use a robust, reliable and reproducible animal challenge model. The difficulty in establishing a lung challenge model relies on choosing an appropriate bacterial strain and administration route, and finding the optimal bacterial dose and challenge timespan that do not lead to either rapid bacterial clearance or severe sepsis and death [31]. Moreover, in consideration of refinement, survival studies involving challenges with lethal bacterial doses are increasingly discouraged [32]. The oropharyngeal aspiration technique is a non-invasive, rapid and technically undemanding method that can reduce the use of mice and improve animal welfare [23,33]. This study demonstrates that it has applications to murine models of acute lung challenge with *P. aeruginosa* or *A. baumannii*, and comparable bioburdens are achieved relative to models established using other bacterial delivery techniques.

We selected the *P. aeruginosa* KK1 strain from the international *P. aeruginosa* reference panel [26] as it is a well-characterized, sequenced respiratory CF early clinical isolate that displays most of the virulence traits of early infecting strains [27,34]. Indeed, KK1 was shown to be the most virulent strain among the three KK clonal isolates in two invertebrate models but less virulent than other early sequential isolates (AA2 and MF1), diminishing the risk of compromising animal welfare [35]. In another murine model, administration of comparable bacterial doses of *P. aeruginosa* AA2 and KK1 strains resulted in higher mortality in mice infected with the former strain [35,36]. Additionally, it was shown that the susceptibility of two mouse strains (BALB and C57BL/6) to KK1 infection was comparable [35]. We chose the *A. baumannii* ATCC 19606 strain as it stands out as the most characterized strain in this species and is frequently used in virulence and vaccine studies. While recent clinical isolates, such as strains LAC-4 and AB5075, typically show more virulence or even hyper-virulence [37], animal models employing these strains usually result in severe infections, posing concerns for animal welfare [38].

We observed that mice were able to clear low doses of the *P. aeruginosa* KK1 strain within 48 h and that the bacterial burden in the organs reached a peak at 24 h. Similarly, a report of 5×10^6^ c.f.u. of PAO1 strain delivered to mice via intrapulmonary administration also showed that the c.f.u. recovery significantly dropped 48 h post-challenge and that bacteria were completely cleared after 72 h [39]. We observed that over a wider dose range (1×10^5^–3×10^7^ c.f.u. per mouse), the bioburden at 24 h increased with the challenge dose and positively correlated with the observed increase in the severity scale. To ensure sufficient bacterial burden in the organs without compromising animal welfare, it was concluded that ≥4×10^6^ c.f.u. per mouse was optimal. These results are in accordance with other *P. aeruginosa* acute lung challenge models utilizing clinical strains but a different delivery technique, which usually administers doses in the 10^6^–10^7^ range, with the infections lasting no longer than 3 days [19]. As mentioned, a key consideration for us was to have a reproducible bacterial burden that allowed us to reliably distinguish effectively between mice that have been treated with a vaccine or therapy versus control mice without the animals showing signs of being sick. Higher or lower bacterial doses could also be utilized depending on researchers’ requirements and institutional severity bands.

In this study, we also present an optimized mouse acute lung challenge model using the non-invasive oropharyngeal aspiration technique to deliver *A. baumannii* ATCC 19606 into female C3H/HeN mice at a dose ranging from 1×10^7^ to 9×10^7^ c.f.u. 50 µl^−1^ per mouse. Substantial bacterial burdens were maintained in the lungs, with 5- to 7-log_10_ c.f.u. g^−1^ at 24 h post-challenge and 2- to 4-log_10_ c.f.u. g^−1^ at 48 h. The obtained results aligned with previously reported studies. Specifically, when infecting mice with 10^7^ c.f.u. of non-hypervirulent *A. baumannii* strains, previous studies have noted bacterial burdens in the lungs ranging from 3- to 5-log_10_ c.f.u. ml−1 at 24 h and 2- to 4-log_10_ c.f.u. ml−1 at 48 h, accompanied by mild-to-moderate clinical signs of infection [38,40, 41]. While the original tracheotomy model observed organ colonization of 3-log_10_ c.f.u. per organ 14 days post-infection [30], we did not detect bacterial burden after 7 days using oropharyngeal aspiration (data not shown). This difference suggests that, when employing the oropharyngeal aspiration technique, mice may experience improved recovery post-bacterial instillation without surgical trauma, and higher bacterial doses may be necessary to establish long-term infection and would require further optimization. A recent study reported that the oropharyngeal and surgical intratracheal instillation methods generated comparably robust and consistent infections within the lower respiratory airways of neutropenic mice. Both instillation methods reported comparable levels of bacterial colonizations, pulmonary lesions, leucocyte infiltration and inflammatory biomarkers [42]. While we have not compared both methods directly, our current study supports this and further advocates for the more refined oropharyngeal instillation over surgical intratracheal instillation.

To date, only two studies utilizing oropharyngeal instillation to develop acute pneumonia in mice with *P. aeruginosa* have been published. These were reported on the instillation of 10^5^ c.f.u. of PA103 strain and 10^7^ c.f.u. of MB447 strain and proved to be useful for the evaluation of a prophylactic vaccine and antimicrobial compounds [43,44]. The number of reports is surprisingly low, given its reproducibility and lack of lethality, and it does not require any surgical procedure. Similarly, only a limited number of studies have employed this technique to induce lung infections in mice with *A. baumannii* compared to the intratracheal route. These studies utilized inoculations ranging from 10^7^ to 10^8^ c.f.u. of the ATCC 17978 strain or clinical isolates, demonstrating effectiveness in establishing a pneumonia model [43,44]. Notably, this approach is considered to better replicate the droplet transmission observed in healthcare-acquired pneumonia. Using this model allows for a more accurate examination of the pathogenesis of pneumonia-causing pathogens and the evaluation of new therapeutic interventions, offering a closer approximation to the aspiration pneumonia seen in humans.

We monitored stomach bioburden in both models to assess what we initially considered to be contamination of the stomach in the method. However, it should be noted that the bioburden of *P. aeruginosa* in the stomach may be due to dissemination, at least in part. Comparison of the proportional *P. aeruginosa* bacterial burden in the stomach is typically 1–2 % once proficiency had improved (example in Fig. S1), while the stomach bioburden in *A. baumannii*-challenged mice was much lower, and is typically 5 log c.f.u. lower in the stomach than in the lung (Fig. S1). Both sets of instillations shown in Fig. S1 were performed by the same individual within a relatively short period of time (weeks); therefore, the difference in stomach burden shown is unlikely to be due to technique and more likely strain related.

Overall, this non-invasive mouse challenge model offers a valuable tool to examine the potential of therapies against *P. aeruginosa* or *A. baumannii* infections with improved adherence to the 3Rs principle [25]. Uniform bilateral distribution of the bacteria in the lungs was achieved at reproducible c.f.u. in 24 h for both species. Such findings underscore the equivalent reliability and effectiveness of the oropharyngeal aspiration method for establishing *A. baumannii* lung infections in mice, offering a less invasive alternative to tracheotomy models. This easy-to-perform model will enhance the development of therapeutic interventions and vaccines targeting these challenging organisms.

## supplementary material

10.1099/acmi.0.000860.v3 Uncited Supplementary Material 1.

## Abbreviations

CF: cystic fibrosis
c.f.u.: colony forming units
IMS: industrial methylated spirit
LB: Luria-Bertani
MGS: mouse grimace scale
MP: methylated spirit
OD: optical density
PBS: phosphate-buffered saline
RTIs: respiratory tract infections
RTIs: respiratory tract infections
SD: standard deviation
SEM: standard error of the mean
UK: United Kingdom
US: United States
