# Supplementary material for: Development of acute Pseudomonas aeruginosa and Acinetobacter baumannii lung mono-challenge models in mice using oropharyngeal aspiration
Source: Access Microbiol. 2024 Nov 21;6(11):000860.v3. doi: 10.1099/acmi.0.000860.v3 (PMC11580749; doi:10.1099/acmi.0.000860.v3)
Supplement: Uncited Supplementary Material 1. [file acmi-6-00860-s001.pdf]

Development of acute *Pseudomonas aeruginosa* and *Acinetobacter baumannii* lung mono-challenge models in mice using oropharyngeal aspiration.

Irene Jurado-Martín*,[1,2] Chaoying Ma*,[1,2] Nouran Rezk[1,2], Maite Sainz-Mejías,[1,2] Yueran Hou,[1,2] John A. Baugh[2,3] and Siobhán McClean[1]

[1] School of Biomedical and Biomolecular Science, University College Dublin, Dublin, Ireland

[2] UCD Conway Institute of Biomolecular & Biomedical Research, University College Dublin, Dublin, Ireland

[3] UCD School of Medicine, University College Dublin, Belfield, Dublin, Ireland

* Both these authors contributed equally to this work.

Corresponding author: siobhan.mcclean@ucd.ie

SUPPLEMENTARY MATERIAL

Table S1. Scoresheet used to evaluate mice severity after each procedure.

COMBINED POST PROCEDURAL / TRIAL SCORE SHEET (General & MGS combined) 07.10.20			
PI / Researcher		Siobhán McClean/Irene Jurado	Animal ID:- Treatment Group:-
HPRA Project		AE18982/P209	
SCORE SHEET			Date/Time / Initials
Appearance	0	Normal	
	1	Slight piloerection / slight change in posture or respiration	
	2	Moderate piloerection / moderate change in posture or resp	
	3	Pronounced piloerection / pronounced change in posture or resp	
Weight		Initial weight (g)	
Weight change	0	Normal	
	1	Body wt <10% decrease	
	2	Body wt 10-20% decrease	
	3	Body wt >20% decrease	
Behaviour	0	Normal	
	1	Minor depression	
	2	Decreased mobility or alertness/isolated	
	3	Noticably still / Precomatose / Restless / Vocalisation	
Hydration	0	Normal	
	1	5% dehydration - skin tents briefly, but returns to normal	
	2	10% dehydration - Skin tents and takes more than a few seconds to return	
	3	15+% dehydration - skin tents and stays tented	
		Total (0-12)	
MOUSE GRIMACE SCALE (MGS)			
Orbital Tightening (0, 1, 2)			
Nose Bulge (0, 1, 2)			
Cheek Bulge (0, 1, 2)			
Ear Position (0, 1, 2)			
		Total	
BINARY OBSERVATIONS - Comments below (with date) if answer is Y or Not OK			
Analgesia administered subsequent to scoring (Y/N)			
Skin Lesions		Notify BMF staff if severe	

SCORE SHEET ACTIONS (FOR MODERATE BANDING)	
Score 0-1	**Normal** - No action required
Score 2-4	**Repeat scoring in 6 hours**
Score 5-7	**Suffering** - Administer analgesia/observe regularly/notify BMF staff (and DV by email)
**Signs of pain** - Analgesia per protocol/notify BMF staff. Euthanasia for severe/intractable pain	
**Animal also to be euthanased (unless DV consulted) if:**	
**a) Score of 3 in any single category or b) Total score of greater than 7 or c) Otherwise indicated**	
COMMENTS / TECHNICAL OFFICER OBSERVATIONS: **Written (with date/time) on the back of the sheet and a tick placed .... here to indicate this has been done.**	

MGS ACTIONS (FOR MODERTE BANDING)	
**Score**	**Actions**
0-2	None
3-4	Administer analgesia and score again in four hours
5-6	Administer analgesia, notify DV by email and score again in four hours -> euthanise at that point if still scoring the same or higher
>7	Immediate euthanasia unless approved by DV to continue on trial. Notify DV by email.

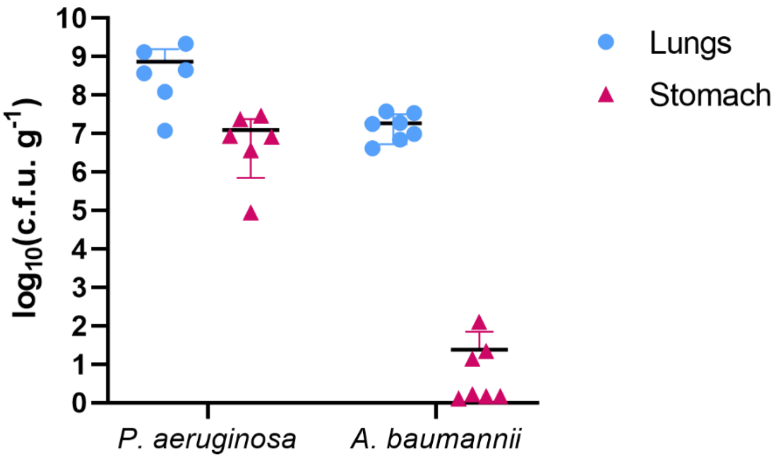

Figure S1. Comparison of bioburden in the lungs and stomach of mice challenged with *P. aeruginosa* or *A. baumannii*. The graph compares the bioburdens obtained from an experiment performed by the same researcher, once optimisation studies were complete. *P. aeruginosa* bioburden is consistently higher than that of *A. baumannii*. Results are represented with the mean ± SEM, with each point representing one mouse.

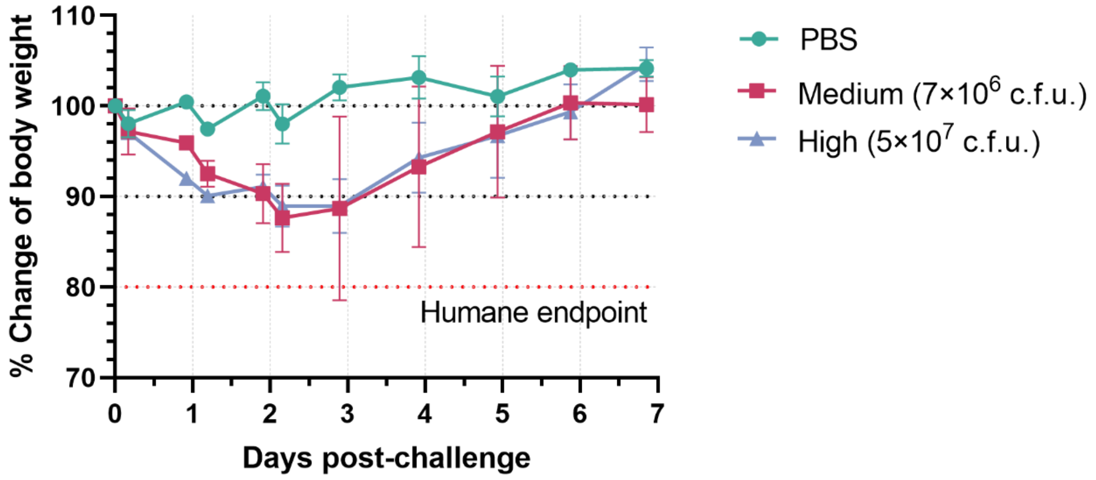

Figure S2. Results of the acute *A. baumannii* pneumonia model 7 days after challenge. Changes in mice weight relative to that before the procedure. Each point represents the mean ± SEM of the mice in the group (n=2).

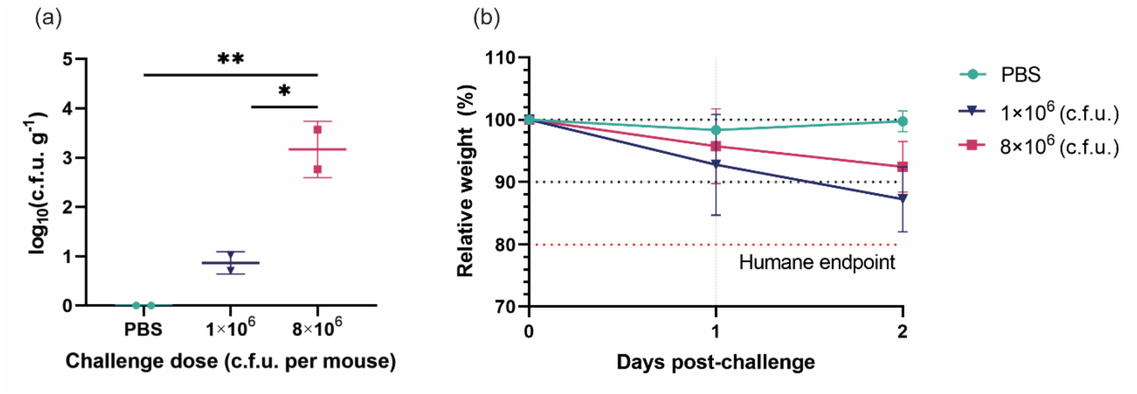

Figure S3. Initial dose-finding pilot study performed acute *A. baumannii* pneumonia model 48 h after challenge. (**a**) Bioburden in the lungs of mice challenged with PBS, low, or medium bacterial doses. Results are represented with the mean ± SEM, with each point representing one mouse. (**b**) Relative weight lost or gained by mice relative to that before the procedure. Each point represents the mean ± SEM of the mice in the group (n=2).
